# Directional selection, not the direction of selection, affects telomere length and copy number at ribosomal RNA loci

**DOI:** 10.1038/s41598-024-63030-x

**Published:** 2024-05-28

**Authors:** Daniel E. Sadler, Phillip C. Watts, Silva Uusi-Heikkilä

**Affiliations:** Department of Biological and Environmental Science, University of Jyväskylä, 40014 Jyväskylä, Finland

**Keywords:** Experimental evolution, Genetic markers, Evolutionary biology, Telomeres

## Abstract

Many fisheries exert directional selection on traits such as body size and growth rate. Whether directional selection impacts regions of the genome associated with traits related to growth is unknown. To address this issue, we characterised copy number variation in three regions of the genome associated with cell division, (1) telomeric DNA, (2) loci transcribed as ribosomal RNA (rDNA), and (3) mitochondrial DNA (mtDNA), in three selection lines of zebrafish reared at three temperatures (22 °C, 28 °C, and 34 °C). Selection lines differed in (1) the direction of selection (two lines experienced directional selection for large or small body size) and (2) whether they experienced any directional selection itself. Lines that had experienced directional selection were smaller, had lower growth rate, shorter telomeres, and lower rDNA copy number than the line that experiencing no directional selection. Neither telomere length nor rDNA copy number were affected by temperature. In contrast, mtDNA content increased at elevated temperature but did not differ among selection lines. Though directional selection impacts rDNA and telomere length, direction of such selection did not matter, whereas mtDNA acts as a stress marker for temperature. Future work should examine the consequences of these genomic changes in natural fish stocks.

## Introduction

Many fisheries are size-selective and remove the largest individuals within a population. Such directional selection on body size can elicit marked phenotypic changes, such as a reduced growth rate and age at maturation^1–4^. Experimental studies have shed light on the phenotypic^2,5^ and genomic^6^ effects of size-selective harvesting by comparing the effects of selection favouring small and large body size against lines of fish that have been harvested at random with respect to body size (hereafter called ‘random-selected’). Random-selection controls for any effects of loss of genetic diversity that accompanies the harvesting process per se. Directional selection on body size elicits significant differences in adult body size and juvenile growth rate between lines selected for small and large body size, whereas random-selected fish can reach similar adult body sizes as large-selected fish^2^ (but see^5,7^). As traits such as size and growth rate are important fitness components^8^, an important issue for fisheries management is to mitigate against effects of directional selection on body size by, for example, implementing alternative fishing strategies.

Unlike size-selective harvesting, balanced harvesting does not target any particular body size^9^ and is thus hypothesised to mitigate the effects of directional selection on exploited fish populations^10^ by maintaining phenotypic diversity, but the efficacy of balanced harvesting is little known^11^. An alternative mitigation action is a moratorium where cessation of fishing should allow phenotypic and genetic recovery^12^. The outcomes of some experimental studies indicate that a moratorium could lead to phenotypic recovery after 12 generations (which was three times longer than the harvesting period of four generations^12,13^). In our experimental selection lines of zebrafish, differences in body size and growth rate between small- and large-selected lines (i.e. that experienced directional selection) induced by five generations of harvesting eroded after ten generations without harvesting (see^14^, and methodology). Interestingly, the random-selected zebrafish had significantly higher growth rate and attained a larger adult body size than either small- or large-selected fish. The apparent convergence between the small- and large-selected lines may imply that some general feature of directional selection, rather than the direction of selection itself, has an effect on body size and growth (and associated traits). Furthermore, as the legacy of directional selection impacted growth rate and body size, it is reasonable to expect a difference among lines in genomic regions that are sensitive to cell division (i.e., growth), such as telomeres, loci that are transcribed as ribosomal RNA, and mitochondrial DNA.

Telomeres are located at the ends of linear (eukaryotic) chromosomes^15^, which in many vertebrates comprise tandem repeats of the motif TTAGGG that are usually about 5–15 kb long^16^. Cell senescence is triggered when a certain proportion of a cell’s telomeres become critically short^17,18^ and telomere length predicts fitness traits in some animals^19–22^. Telomeres shorten with cell division^23^ unless the telomeres are repaired, for example by telomerase ^24,25^.

Loci that are transcribed as ribosomal RNA (hereafter referred to as rDNA) are comprised of tandem arrays of the rRNA cassette (18S, 5.8S, and 28S rRNA loci). Transcription of rDNA is necessary for ribogenesis and protein synthesis. Yet unequal recombination of rDNA can generate a change in rDNA copy number^26^. Indeed, rDNA copy number is sensitive to cell division, as cell division can cause rDNA to become unstable, stimulating molecular aging signals, accelerating cell death, and increasing cancer risk^26^. Interestingly, rDNA exhibits marked intraspecific variation ^27–31^, and this may be relevant for adaptation or as a biomarker of health as variation in rDNA copy number apparently regulates gene expression^29,32,33^ and affects genome stability^26,34–36^. While the association between rDNA copy number and growth rate is less studied than the association between growth and telomere length, it has been shown that rDNA copy number negatively correlates with body mass in humans and rats^37^.

Mitochondria are membrane-bound organelles that contain their own genome (mitochondrial DNA, mtDNA) and which have essential metabolic and cell-signalling roles, notably by supplying most of a cell's energy requirements^38^. By varying the rate of synthesis and degradation^39^, mitochondrial density in cells (and thus mtDNA content) can vary with age^40^ and growth rate^41^. mtDNA depletion can be indicative of cellular malfunction and/or disease ^42,43^. It is relevant to quantify mtDNA content in tandem with variation in telomere length and rDNA copy number as (1) mitochondrial content is coupled with rDNA copy number, at least in humans^29^, and (2) mitochondria are a prime source of intracellular reactive species (ROS) (Murphy 2009) that can damage telomeres^44–47^.

An important feature of these three regions of the genome is that they are sensitive to environment stress. Short telomeres and/or an elevated rate of telomere attrition is a common feature of exposure to stress^44–46,48,49^. Likewise, instability in rDNA copy number has been associated with exposure to environmental stress^33,50–53^ and mtDNA content varies with exposure to pollutants^54,55^. It is therefore relevant to ask whether processes that likely impact copy number, such as directional selection on body size that alters growth, have a concomitant effect on copy number when organisms experience environmental stress. The interaction between environment stress and directional selection induced by fisheries is notoriously difficult to study in nature and thus far only few studies have assessed such interaction^56,57^.

An important environmental stress is thermal stress. For example, extreme weather events can raise sea surface temperature by 2–4 °C, and sometimes > 5 °C^58,59^. Also, temperature regime may affect telomere length, rDNA copy number, and/or mtDNA dynamics, as temperature influences growth rate (i.e. cell division) in many taxa, including teleost fish^60–62^. Indeed, temperature effects on telomere length have been reported in teleosts; for example Siberian sturgeon (*Acipenser baerii*) exhibited a 15% reduction in relative telomere length (RTL) when exposed to 3 °C above ambient^63^, whilst mosquito fish (*Gambusia holbrooki*) had shorter telomeres at lower temperatures^64^.

Our zebrafish (*Danio rerio*) selection lines provide an excellent model to determine whether phenotypic differences in body size and growth between lines of fish that experienced directional selection (small and large size) and non-directional selection (random size) associate with differences in telomere length, rDNA copy number, and/or mtDNA content. We also ask whether any differences among lines affect the genomic response to temperature. To address these questions, we exposed young zebrafish (age 50 days) from three selection lines: (1) small-selected fish experiencing directional selection for small body size, (2) large-selected fish experienced directional selection for large body size, and (3) random-selected fish experienced no directional selection, but the lines nonetheless experienced the same reduction in population size during harvesting (see Methods and^2^ for more details) to ambient (28 °C), low (22 °C) or elevated (34 °C) temperatures. We hypothesised that (1) directional selection would reduce telomere length and copy number of rDNA and mtDNA compared with random-selection, and (2) that thermal stress would also reduce telomere length and copy number. Furthermore, we predicted that (3) there would be an interaction between thermal stress and directional selection, such that fish exposed to directional selection experience more drastic changes in telomere length and copy number under thermal stress.

## Results

**Figure 1:**
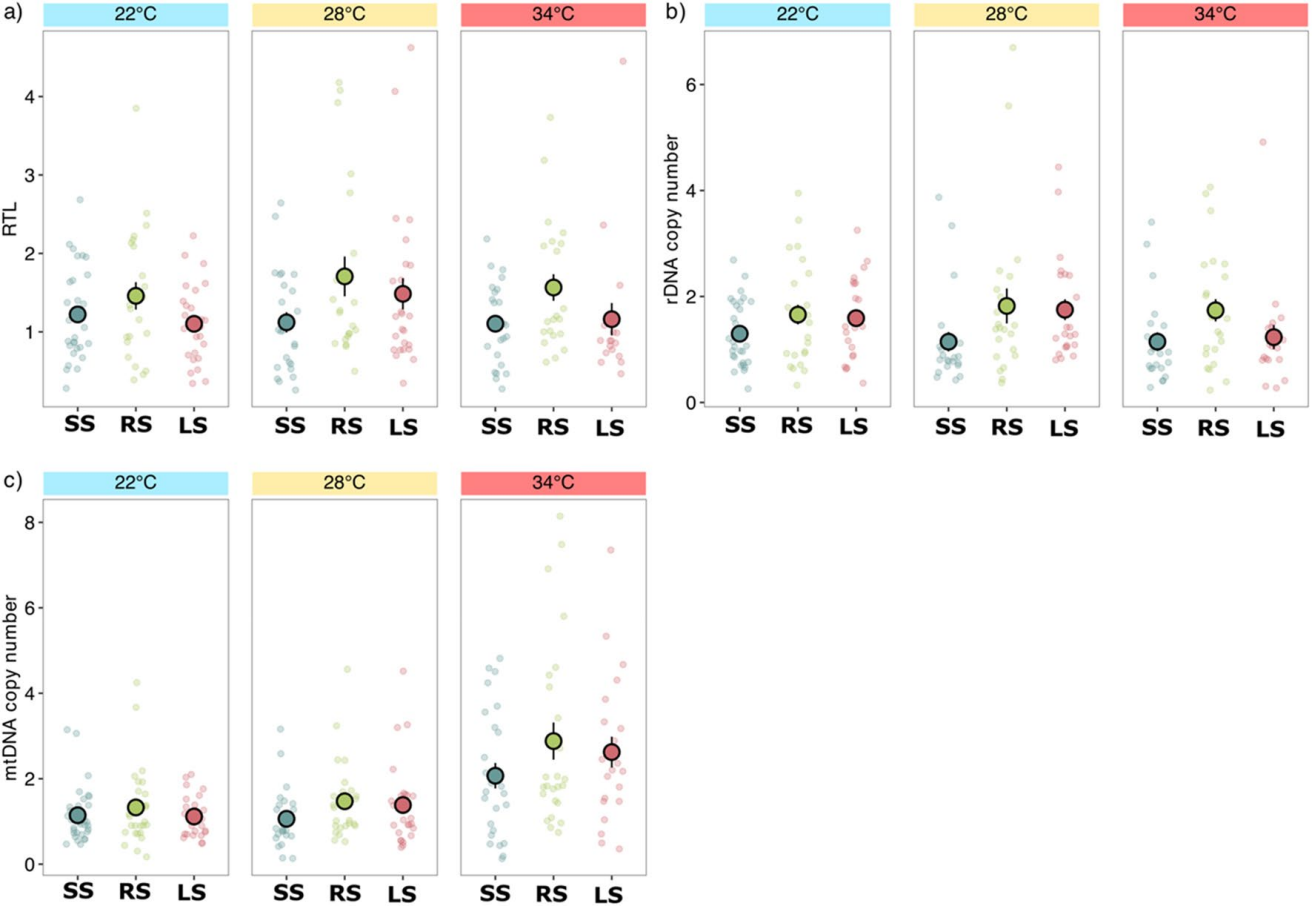
Variation in (**a**) relative telomere length (RTL), (**b**) rDNA copy number, and (**c**) mtDNA content among three size-selection lines: large-selected (LS), random-selected (RS), and small-selected (SS) reared for 250 days at three temperatures (22 °C, 28 °C, and 34 °C). Data are shown as individual observations per fish (small circles) and as the mean (large circles) with standard errors. Within each treatment combination.

Random-selected fish had significantly higher relative telomere length than either small- or large-selected lines (F_2, 247_ = 7.09, *p* < 0.001; Fig. 1a, Table S6). Such differences in telomere length suggests a general effect of directional selection on body size on telomere length rather than any specific effect of the direction (for large or small body size) of selection. Temperature treatment had no significant effect on telomere length, neither was the interaction between selection line and temperature treatment significant in these regions (Table S6).

Similar to relative telomere length, rDNA copy number differed among the three selection lines: random-selected fish had a higher rDNA copy number than fish from either of the size-selected lines, which similarly suggests a general influence of directional selection on rDNA copy number (F_2, 242_ = 7.92, *p* < 0.001; Fig. 1b; Table S6). Also similar to relative telomere length, the temperature treatment did not have a significant effect on rDNA copy number (Table S6), and nor was there a significant interaction between selection line and temperature (Table S6).

In contrast to the pattern of variation in relative telomere length and rDNA copy number, mtDNA content did not significantly differ among the selection lines (Table S6). However, mtDNA content was higher in zebrafish reared at the elevated temperature compared with fish maintained at the low and ambient temperatures (F_2, 262_ = 20.2, *p* < 0.001; Fig. 1c, Table S6). The interaction between the selection line and temperature treatment was not significant (Table S6).

There were significantly positive correlations between relative telomere length and rDNA copy number in all three experimental temperatures (r = 0.366, *p* < 0.001; Figure S1a, b). A significantly positive correlation between telomere length and rDNA was also observed between all selection lines (r = 0.366, *p* < 0.001; Figure S1a, b). mtDNA content was negatively correlated with relative telomere length and rDNA copy number in the large-selected line across the three different temperature treatments (mtDNA:rDNA, r = − 0.263, *p* < 0.05; mtDNA:RTL, r = − 0.256, *p* < 0.05; Figure S1a). mtDNA was negatively correlated with rDNA copy number at 22 °C across all three selection lines (r = − 0.230, *p* < 0.05; Figure S1b).

## Discussion

Size-selective harvesting impacts diversity at single-copy regions of the genome such as microsatellite loci^7^ or SNPs within and among protein-coding regions^6,65^. However, whether size selective harvesting elicits other types of genomic change, such as variation in copy number, and whether any genomic changes affect the response to temperature is not known. Using an experimental zebrafish model, we found that directional selection (for small and large body size) associates with a reduction in telomere length and rDNA copy number, but has no significant effect on mtDNA content, compared to random-selection (i.e., no directional selection). Hence, relative telomere length and rDNA copy number exhibited a correlated response to directional selection, rather than the direction of selection, per se. While mtDNA content was not impacted by directional selection, fish reared at an elevated temperature exhibited an increase in mtDNA content. Counter to our hypothesis we found no evidence of an interaction between directional selection and thermal stress at any of the genomic regions, suggesting an independent action of these processes on copy number variation and telomere length.

Short relative telomere length associating with directional selection on body size is intriguing as, after ten generations of recovery, both directionally selected lines had lower growth rate and reached smaller adult body size than fish which had not experienced directional selection^14^. As telomeres shorten with cell division, unless repaired by telomerase^66^, fast growing, larger individuals (random-selected fish) are expected to have shorter telomeres. Fast growing transgenic coho salmon (*Oncorhynchus kisutch*) are unable to maintain telomere length^67^, and in brown trout (*Salmo trutta*), body size (but not compensatory growth) was negatively associated with telomere length^68^ and a greater change in telomere length^21^. However, telomere length and expression of telomerase increase with development in zebrafish muscle such that telomeres do not shorten with growth in healthy zebrafish until old age (about 30 months) when telomerase expression declines^69,70^. It could be speculated that the fish under directional selection were less capable of telomere maintenance than the line that experienced random selection, however the mechanisms for this would require further studies.

Loss of genetic diversity^71–74^, and potentially inbreeding^75,76^, is a feature of many overharvested fish stocks. Directional selection may elicit a faster loss of genetic diversity than expected under a population reduction alone^77^, as favouring a specific phenotype (e.g., body size) can cause directional shift in allele frequency^78^. Although we do not measure inbreeding or genetic diversity, slower growth associated with directional selection may indicate that these lines experience some inbreeding depression whose effects extend to telomere maintenance^14^. However, the relationship between telomere length and inbreeding is controversial. Studies on wild vertebrate populations have shown that inbreeding/elevated levels of homozygosity is associated with short^79,80^ and long telomeres^81^, or have failed to uncover any significant effect of inbreeding on telomere length^82^. Nonetheless, the comparably short telomeres in both size-selected lines indicates that directional selection, at least for body size, can have an unintended, but important outcome on telomere length—an effect that has not been reported in teleost fish.

As it is not possible to quantify changes in telomere length using non-destructive sampling (e.g. from blood^83^) on such young zebrafish, we do not know whether the outcome of our experiment reflects an inherent difference in telomere length among the lines or whether all fish had similar length telomeres at hatching and the shorter telomeres are a consequence of poor telomere maintenance in the size-selected lines. Nonetheless, that size-selective harvesting can cause short telomeres and/or poor telomere maintenance is a potential cause for concern for the health of overharvested fish stocks given the widespread reports that short telomeres are a biomarker for stress exposure or reduced health in many animals^19–22,84,85^ including telomerase deficient zebrafish^86^.

Variation in temperature did not impact telomere length in zebrafish in contrast with previous studies demonstrating a negative association between water temperature and telomere length in brown trout (*Salmo trutto*)^21,68^. In sticklebacks (*Gasterosteus aculeatus*), variation in temperature did not directly affect telomere length at individual level but had sex specific effects on telomere length in mature fish^87^. This is interesting as old (> 18 months) zebrafish have short telomeres^88^, implying that a longer experimental duration might have revealed additional changes in telomere length in our lines. The lack of interaction between selection line and temperature stress on telomere length is surprising following previous interactions on phenotypic traits including growth^56^. But the lack of interaction is relevant if the size-selected lines experience some inbreeding depression, because a greater impact of inhabiting a poor environment on telomere length was uncovered in less genetically diverse juvenile birds^79^.

As rDNA copy number is sensitive to environment variation^33,50,52,89^ it represents a hypothesised ‘environmental sensor’ that may regulate the molecular response to environmental cues^35,47,90,91^. It is therefore surprising that we found no significant impact of different temperature environments on zebrafish rDNA copy number. Future work could study different species and/or different environmental stressors, such as nutrient stress^92^ or exposure to pollutants^52^, to uncover possible drivers of rDNA copy number variation in teleosts. Nonetheless, it is interesting that directional selection on body size affected rDNA copy number as significant differences in rDNA copy number among strains of inbred laboratory mice (*Mus musculus*^93^) raise the prospect that rDNA copy number might be impacted by reductions in population size/inbreeding (as discussed for telomere length). Indeed, the significant positive correlation between rDNA and relative telomere length supports the idea that these regions are sensitive to similar stressors^94^. For example, rDNA and telomeres are both sensitive to changes in heterochromatin architecture^95^ and oxidative stress^94^. Our data highlight a need to quantify rDNA copy number and telomere length together to determine in what taxa, in what environments, and potentially why, copy number/length of these regions of the genome are co-associated. Moreover, it is important to understand these changes in the context of traits associated with growth as these regions are sensitive to cell division^23,26,66^. The negative association between mtDNA content and rDNA copy number in zebrafish is consistent with the negative association between these regions of the genome in humans^29^. However, the weak relationships between mtDNA content and rDNA copy number likely reflects that mitochondria have a separate genome and mitochondrial density being dynamic and independent of cell division^96^.

The significant effect of temperature on mtDNA content at 34 °C adds to the diversity of biological impacts that occur in aquatic communities exposed to thermal stress. To our knowledge, only one previous study has examined the effect of temperature on mtDNA content in teleosts, in which there was an increase in mtDNA content in eggs at warmer winter temperatures (+ 5 °C) in stickleback (*Gasterosteus aculeatus*)^97^. Similarly, an increase in mtDNA content was reported in prawns (*Palaemon carinicauda*) raised in warm water^98^. An increase in mtDNA content corresponds with an increase in mitochondrial content^99^ and is consistent with an expected rise in metabolic rate that accompanies an increase in temperature^100,101^. Studying mtDNA content in tandem with telomere length and rDNA copy number is relevant as mitochondrial density may positively associate with the production of reactive oxygen species^102–104^ that can damage telomeres^44–46^ and rDNA^105^. For example, an increase in mtDNA density was correlated with production of the free radical superoxide, which in turn influenced telomere length^103^. Indeed, we found negative correlations between mtDNA content and telomere length/rDNA copy number in the large-selected lines, which (1) reinforces the idea that telomere length (and rDNA copy number) should be quantified in tandem with mtDNA content, and ideally ROS production^103^, and (2) shows how any association between these regions of the genome can depend on genetic background.

Balanced harvesting is hypothesised to mitigate the effects of directional selection^10^, for example by retaining genetic diversity and lessening any effects of inbreeding^11^. Balanced harvesting can increase stock productivity^106^, aid the recovery of a stock’s natural size and age structure^107^, and improve the resilience of a stocks to natural disturbance^108^. Here, our random-selected lines experienced harvesting but no directional selection on body size and thus correspond with balanced harvesting. Our zebrafish model of overharvesting supports the idea that balanced harvesting can help maintain growth and body size. We also show how harvesting regime can impact regions of the genome that are associated with organismal health and fitness^19–22^.

We show that directional selection (for body size) has a greater impact on these regions of the genome than an equivalent random reduction in population size. Identifying the mechanisms behind these results requires further work but may be related to a loss of genetic diversity that accompanies directional selection. Quantifying processes that drive variation in telomere length and rDNA copy number is important as maintenance of these loci is thought to be essential to genome integrity^35,109,110^ and the rate of molecular aging^89,111^. Intriguingly, our data indicate relative telomere length and rDNA copy number are resilient to changes in temperature. In contrast, mtDNA content was not impacted by directional selection but was increased at elevated temperatures, presumably in response to a change in metabolic requirements. Our data open new avenues for future research of dynamics of telomere length, rDNA copy number, and mtDNA content in wild populations. For example, a next step would be to determine whether natural populations of exploited fish experienced similar genomic impacts and, if so, what are the mechanisms and do these genomic changes alter individual fitness. Crucially, direction of selection (either small- or large-selection on body size) appears less important that the act of directional selection itself, as directional selection reduced rDNA copy number and telomere length regardless of the direction compared to random selection. Our data suggest that selection regimes implicated by fisheries should be reconsidered, utilising alterative harvest strategies such as balanced harvesting to reduce any effects of directional selection on fitness.

## Methods

### Zebrafish model system

Three zebrafish selection lines (two replicates of each line) were created by subjecting wild-caught fish (from the West Bengal region of India^112^) to the following harvesting regimes: (1) small-selection, where 75% of the largest fish were removed, (2) large-selection in which 75% of the smallest fish were removed, and (3) random-selection (population loss alone), where 75% of the fish were removed at random with regard to body size. After five generations of harvesting, small-selected fish were smaller, had higher juvenile growth rate and higher reproductive investment than large-selected fish^2^. After ten generations of no-harvesting (a ‘moratorium’), the random-selected fish had a 12% faster growth rate than both the large- and small-selected lines (which had similar growth rates)^14^. In our model there is a contrast between the large- and small-selected lines that both experienced directional selection on body size in contrast to the random-selected line that experienced the same population reduction (75% harvesting) but no directional selection on body size. All methods were performed in accordance with the relevant guidelines and regulations. All experimental protocols were approved by the Finnish Project Authorisation Board. Licence no. ESAVI/24,875/2018 and all experiments followed the ARRIVE guidelines^113^.

### Study design

Fish (*n* = 243; Table S2) at age 50 days post fertilization were taken from each selection line replicate and exposed to three different temperatures, low (22 °C), ambient (28 °C), and elevated (34 °C), for 250 days. Ambient temperature is the control temperature, as it was the standard rearing temperature in the laboratory for 15 generations as well as representing the natural environment of zebrafish^114^. Elevated and low temperatures were ± 6 °C from ambient, as this presents a physiological stress to zebrafish^115,116^ and represents a potential rise in water temperature during extreme weather events^59^.

Fish were housed in 30L glass aquaria with three tanks per temperature treatment. Each aquarium housed eight cylindrical mesh cages with five fish in each. Fish were first acclimated for two weeks (28 °C), after which, the temperature was altered by plus or minus 1 °C per day for six days. At the end of the experiment, fish were euthanized with 2-phenoloxyethanol and stored at -20 until DNA extraction.

### qPCR to estimate telomere length and copy number

DNA was extracted from muscle tissue using a DNeasy blood and tissue kit (Qiagen). DNA quantity was measured using a NanoDrop spectrophotometer (ThermoFisher) and samples were normalised to 5 ng/μl. Relative copy number (analogous to relative telomere length; Cawthon 2002) of each sample was calculated by quantitative PCR (qPCR) using an appropriate locus-specific primer pair and a single copy gene (SCG) (see^117^ for relative telomere length^52^, for rDNA, and^55^ for mtDNA RCN).

qPCRs were completed on CFX96 thermal cyclers (Bio-Rad) with each qPCR containing 20 ng DNA, 0.3uM of each primer, and 10 μl of iQ SYBR Green supermix (Bio-Rad). Samples were run in triplicate to provide a mean Ct value if the standard deviation (SD) was < 0.2. If a sample qPCR SD was > 0.2, a mean Ct was taken from two qPCRs, or the qPCR was redone in triplicate if the 0.2 SD threshold was not met. Each qPCR plate (that used the same template; Table S3) contained a negative (no DNA) control, the same ‘golden standard’ DNA (GS), and a serial DNA dilution to calculate qPCR efficiency (1:2 dilution starting from 80 ng/μl). qPCRs were completed on separate plates for the same 26 samples to estimate reproducibility which was high based on Ct values (r > 0.9, *p* < 0.001 for all loci; Table S4). Full details of the qPCR primers (for each locus and for the SCG), and the thermal cycling conditions are provided in Table S5. Relative copy number (RCN) (or relative telomere length, RTL) were calculated for each sample using:

$$RCN(orRTL) = E(target)^{(CtGS - CtSAMPLE)} /E(control)^{(CtGS - CtSAMPLE)} ,$$

where E(target) and E(control) are the qPCR efficiencies of the target (i.e., telomere, rDNA, and mtDNA) and the single copy gene respectively, and C_t_^GS^ and C_t_^SAMPLE^ are the critical cycle thresholds for the golden standard and sample DNAs respectively^117,118^.

### Statistical analysis

All statistical analyses were performed in R studio using R v.4.2.2^119^. We used linear mixed models (LMM) models to assess how relative telomere length, rDNA copy number and mtDNA content differed among selection lines and temperature treatments, whereby temperature, selection line and their interaction were included as fixed terms using the following model:

$${\text{RCN}}\sim {\text{ temperature }}*{\text{ selection line }} + \, \left( {{1 }|{\text{ selection}} - {\text{line replicate}}} \right),$$

where RCN indicates relative telomere length, rDNA copy number, or mtDNA content, and selection-line replicate (*n* = 2 replicates for each selection line) is a random term. Analyses used the *lmer* and *lmertest* functions within lme4 v.1.1-33 and lmerTest v.3.1-3 packages^120,121^. Pearson’s correlation was assessed between pairs of relative telomere length, rDNA copy number, and mtDNA content within each temperature treatment using the *cor.test* function within GGally v.2.1.2^122^.

### Supplementary Information

Supplementary Information.

## Data Availability

Data available in the manuscript supplementary material and from the corresponding author upon reasonable request.
