# Supplementary material for: Directional selection, not the direction of selection, affects telomere length and copy number at ribosomal RNA loci
Source: Sci Rep. 2024 May 28;14:12162. doi: 10.1038/s41598-024-63030-x (PMC11130246; doi:10.1038/s41598-024-63030-x)
Supplement: Supplementary file 1 — Supplementary Information. [file 41598_2024_63030_MOESM1_ESM.docx]

**Supporting information for**

**“****Directional selection, not the direction of selection, affects telomere length and copy number at ribosomal RNA loci**

Authors: Daniel E. Sadler, Phillip C. Watts, Silva Uusi-Heikkilä

**Page Contents**

S2 Supplementary methods

S3 Table S1: LMM summaries for growth rate and body szie

S3 Table S2: Summary of sample sizes

S4 Table S3: qPCR plate layout

S4 Table S4: Reproducibility of primers

S5 Table S5: List of primers used

S5 Table S6: Summary of LMMs for biomarkers

S6 Figure S1: Correlation plots of biomarkers

**Supplementary Methods**

*Primers*

We used standard vertebrate primers: tel1 and tel2 (Cawthon, 2002) to assess relative telomere length (RTL) and c-fos as the single copy gene (Moore & Whitmore, 2014). To assess rDNA copy number, we used 18S rDNA (18S-F, 18S-R) again using c-fos as the single copy gene. Finally, to assess mtDNA copy number we compared mtDNA (mt-F, mt-R) against a nuclear target (nt-F, nt-R) (Hunter et al., 2010). All primer sequences, amplicon length and efficiencies stated in table S4.

*qPCR conditions*

Relative copy number (RCN) or RTL of each sample was calculated by quantitative PCR (qPCR) on CFX96 thermal cyclers (Bio-Rad). Each plate contained 20ng DNA (DNA standardised to 5ng/ul) , 0.3uM of each primer (Table S3) and 10ul SYBR Green supermix (Bio-Rad). Samples were run in triplicate to provide a mean Ct value if the standard deviation (SD) was <0.2; if a sample qPCR SD was >0.2, a mean Ct was taken from two qPCRs or the qPCR was redone in triplicate if the 0.2 SD threshold was not met. Each qPCR plate (that used the same template (Table S2)) contained a negative (no DNA) control, the same ‘golden standard’ DNA (GS), and a serial DNA dilution to calculate qPCR efficiency (1:2 dilution starting from 80ng/μl). qPCRs were completed on separate plates for the same 26 samples to estimate reproducibility (Table S3) which was high based on Ct values.

Thermal cycling conditions for telomere primers (tel1, tel2) were: 10 min at 95°C, followed by 40 cycles of 95°C for 15 sec and 54°C for 2 min followed by melt curve measurement from 65°C to 95°C. Whilst the thermal cycling conditions for c-fos and 18S rDNA were 3 min at 95°C, followed by 40 cycles of 95°C for 10 sec and 60°C for 30 sec followed by melt curve measurement from 65°C to 95°C. Finally, thermal cycling conditions for ntDNA and mtDNA were: 3 min at 95°C, followed by 40 cycles of 95°C for 10 sec and 60°C (62 for mtDNA) for 30 sec followed by melt curve measurement from 65°C to 95°C.

Relative copy number (RCN) (or relative telomere length, RTL) was calculated for each sample using:

RCN (or RTL) = E(target)^(C_t_^GS^ - C_t_^SAMPLE^) / E(control)^(C_t_^GS^ - C_t_^SAMPLE^),

where E(target) and E(control) are the qPCR efficiencies of the target (i.e., telomere, rDNA, and mtDNA) and the single copy gene primers respectively, and C_t_^GS^ and C_t_^SAMPLE^ are the critical cycle thresholds for the golden standard (GS) and the sample DNAs respectively (Pfaffl, 2001; Cawthon 2002; Jernfors et al., 2021).

**Table S1:** Summary of growth rate and body size LMMs

| Trait | Treatment | Df | f value | *p value* |
| --- | --- | --- | --- | --- |
| Length  *log(Length) ~ log(Week) * Temperature * Selection + (Week \| Cage) + (1\|Line) + (1\|Rep_ID)* | log(Week) | 1 | 302.8156 | **<0.001 ***** |
|  | Temp | 1 | 9.8959 | **0.0022 **** |
|  | Selection | 2 | 1.6065 | 0.2060 |
|  | log(Week):Temp | 1 | 57.1119 | **<0.001 ***** |
|  | log(Week):Selection | 2 | 5.9859 | **0.0026**** |
|  | Temp:Selection | 2 | 1.6395 | 0.1994 |
|  | log(Week):Temp:  Selection | 2 | 4.7955 | **0.0085**** |
| Weight  *log(Weight) ~ log(Week) * Temperature * Selection + (Week \| Cage) + (1\|Line) + (1\|Rep_ID)* | log(Week) | 1 | 330.9379 | **<0.001 ***** |
|  | Temp | 1 | 12.6183 | **<0.001 ***** |
|  | Selection | 2 | 3.4461 | **0.0357*** |
|  | log(Week):Temp | 1 | 99.9635 | **<0.001 ***** |
|  | log(Week):Selection | 2 | 15.9350 | **<0.001 ***** |
|  | Temp:Selection | 2 | 2.7615 | 0.0677 |
|  | log(Week):Temp:  Selection | 2 | 11.3802 | **<0.001 ***** |
| Length (SGR) | Selection | 2 | 1.5981 | 0.2109 |
|  | Temp | 2 | 24.6091 | **<0.001 ***** |
|  | Selection: Temp | 4 | 2.5859 | **0.0463 *** |
| Weight (SGR) | Selection | 2 | 6.6572 | **0.0015**** |
|  | Temp | 2 | 24.9650 | **<0.001***** |
|  | Selection: Temp | 4 | 5.5085 | **0.0045**** |

**Table S2:** Summary of sample size in each line and temperature treatment. Original sample size is n=20 per line replicate per temperature, but samples were excluded due to mortality during experiment, inadequate DNA quality, or plate error in qPCR.

| **Temperature** | **Line** | **n** |
| --- | --- | --- |
| 22 | LS1 | 17 |
|  | LS2 | 16 |
|  | SS1 | 16 |
|  | SS2 | 11 |
|  | RS1 | 15 |
|  | RS2 | 13 |
| 28 | LS1 | 15 |
|  | LS2 | 13 |
|  | SS1 | 15 |
|  | SS2 | 11 |
|  | RS1 | 14 |
|  | RS2 | 11 |
| 34 | LS1 | 10 |
|  | LS2 | 16 |
|  | SS1 | 16 |
|  | SS2 | 11 |
|  | RS1 | 11 |
|  | RS2 | 15 |

**Table S3:** Plate design for qPCR (96 well plate)

| S1 | S1 | S1 | S2 | S2 | S2 | S3 | S3 | S3 | L 1:1 | L 1:1 | L 1:1 |
| --- | --- | --- | --- | --- | --- | --- | --- | --- | --- | --- | --- |
| S4 | S4 | S4 | S5 | S5 | S5 | S6 | S6 | S6 | L 1:2 | L 1:2 | L 1:2 |
| S7 | S7 | S7 | S8 | S8 | S8 | S9 | S9 | S9 | L 1:4 | L 1:4 | L 1:4 |
| S10 | S10 | S10 | S11 | S11 | S11 | S12 | S12 | S12 | L 1:8 | L 1:8 | L 1:8 |
| S13 | S13 | S13 | S14 | S14 | S14 | S15 | S15 | S15 | L 1:16 | L 1:16 | L 1:16 |
| S16 | S16 | S16 | S17 | S17 | S17 | S18 | S18 | S18 | L 1:32 | L 1:32 | L 1:32 |
| S19 | S19 | S19 | S20 | S20 | S20 | S21 | S21 | S21 | NTC | NTC | NTC |
| S22 | S22 | S22 | S23 | S23 | S23 | S24 | S24 | S24 | GS | GS | GS |

**Table S4:** reproducibility of markers across 26 samples, r2 being the correlation of the Ct scores across two plates of the same sample.

| Biomarker | r | P value |
| --- | --- | --- |
| Telomeres | 0.9741 | **<0.001***** |
| rDNA | 0.9684 | **<0.001***** |
| cFos | 0.9341 | **<0.001***** |
| mtDNA | 0.9167 | **<0.001***** |
| ntDNA | 0.9126 | **<0.001***** |

**Table S5:** List of primers used for qPCR

| **Region** | **Primer** | **Sequence** | **Amplicon (bp)** | **Efficiency** | **Reference** |
| --- | --- | --- | --- | --- | --- |
| Telomeres | Tel1 | GGTTTTTGAGGGTGAGGGT  GAGGGTGAGGGTGAGGGT | 76 | 1.517 | Cawthon 2002 |
|  | Tel2 | TCCCGACTATCCCTATCCCT  ATCCCTATCCCTATCCCTA |  |  |  |
| cFos | cFos-F | CAGCTCCACCACAGTGAAGA | 176 | 2.014 | Moore & Whitmore 2014 |
|  | cFos-R | GCTCCAGGTCAGTGTTAGCC |  |  |  |
| mtDNA | mt-F | CAA ACA CAA GCC TCG CCT GTT TAC | 198 | 1.828 | Hunter et al., 2010 |
|  | mt-R | CAC TGA CTT GAT GGG GGA GAC AGT |  |  |  |
| ntDNA | nt-F | ATG GGC TGG GCG ATA AAA TTG G | 233 | 1.852 |  |
|  | nt-R | ACA TGT GCA TGT CGC TCC CAA A |  |  |  |
| rDNA | 18S-F | ATCTGTCAATCCTTTCCG | 238 | 1.655 | Tao et al., 2020 |
|  | 18S-R | GGGGAGTATGGTTGCAAA |  |  |  |

**Table S6:** Summary of LMM results from the model biomarker~temperature*selection + (1|line replicate).

| **Biomarker** | **Treatment** | **Df** | **F Value** | **p value** |
| --- | --- | --- | --- | --- |
| rDNA  *R^2^m=0.0821*  *R^2^c=0.0821* | Temperature | 2 | 1.6176 | 0.2009 |
|  | Selection | 2 | 5.8793 | **0.0033**** |
|  | Temperature:Selection | 4 | 1.1455 | 0.3363 |
| mtDNA  *R^2^m=0.2128*  *R^2^c=0.2282* | Temperature | 2 | 27.7573 | **<0.001***** |
|  | Selection | 2 | 2.0525 | 0.2769 |
|  | Temperature:Selection | 4 | 0.6399 | 0.6345 |
| RTL  *R^2^m=0.0658*  *R^2^c=0.0658* | Temperature | 2 | 0.4199 | 0.6577 |
|  | Selection | 2 | 5.6024 | **0.0043**** |
|  | Temperature:Selection | 4 | 0.9906 | 0.4136 |

*R^2^m=marginal R^2^*, *R^2^c=conditional R^2^*

(a)


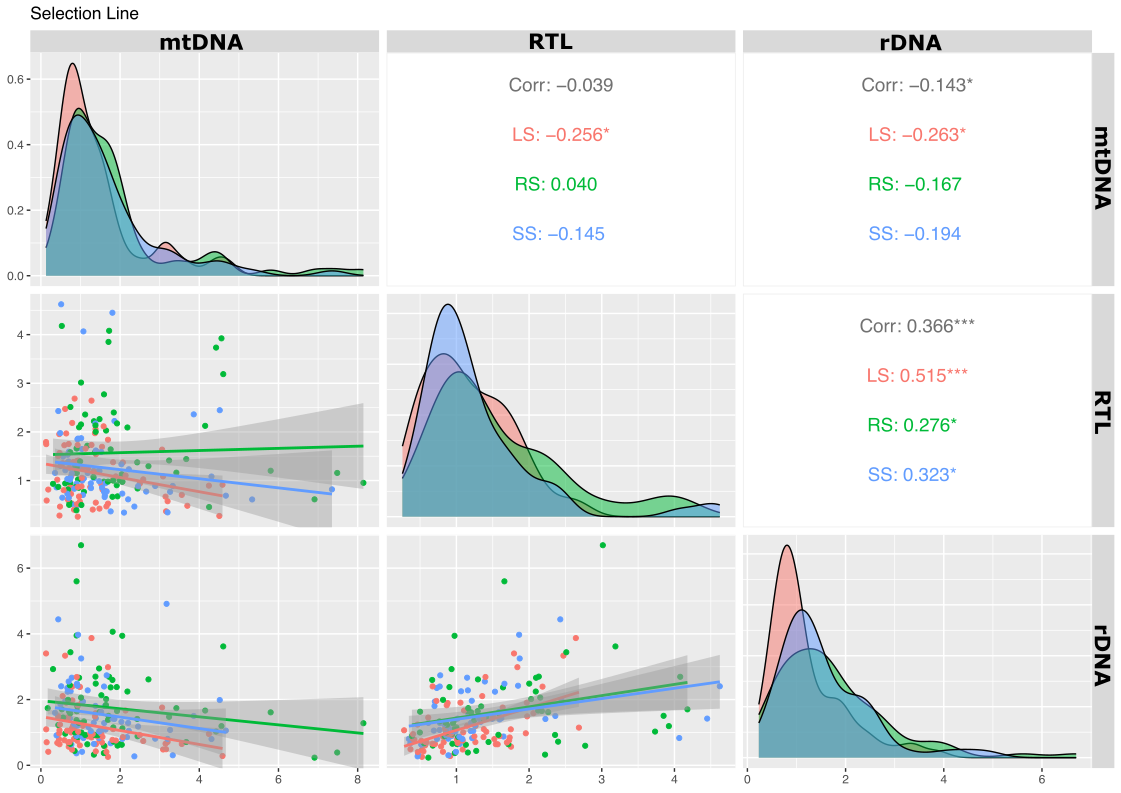


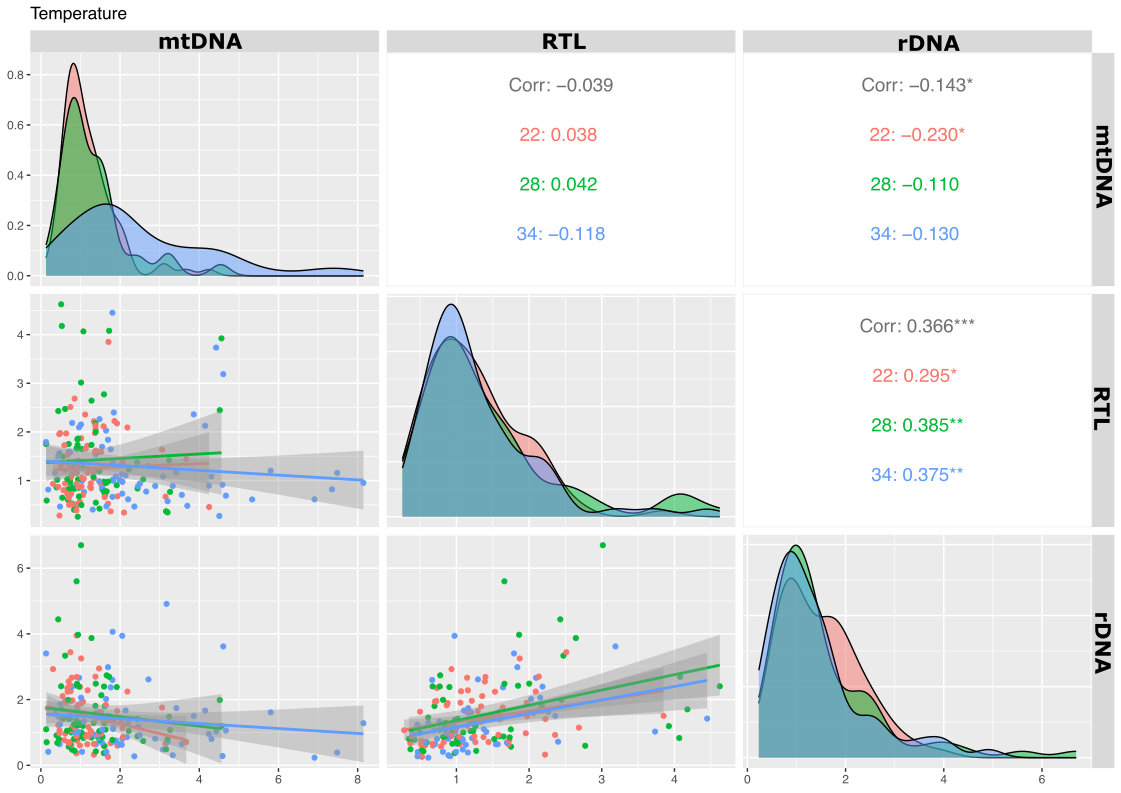


(b)

**Figure S1:** Variation in stress biomarkers amongst (a) selection lines: large-selected (LS), small-selected (SS), and random selected (RS) and (b) temperature treatments (22°C, 28°C, and 34°C). Data shown as density plots and correlation plots with data shown as individual observations per fish (dots). Correlation and significance indicated per treatment interaction with biomarker.
